# Transcriptional Dynamics Induced by Diapause Hormone in the Silkworm, *Bombyx mori*

**DOI:** 10.3390/biology11091313

**Published:** 2022-09-04

**Authors:** Lijuan Chen, Zhongjie Zhang, Kai Chen, Ye Yu, Bo Hu, Hongsheng Song, Xiaojing Liu

**Affiliations:** 1 College of Life Sciences, Shanghai University, Shanghai 200444, China; 2 Jiangsu Key Laboratory of Sericultural Biology and Biotechnology, School of Biotechnology, Jiangsu University of Science and Technology, Zhenjiang 212100, China; 3 Key Laboratory of Silkworm and Mulberry Genetic Improvement, Ministry of Agriculture, The Sericultural Research Institute, Chinese Academy of Agricultural Sciences, Zhenjiang 212100, China; 4 CAS Key Laboratory of Insect Developmental and Evolutionary Biology, CAS Center for Excellence in Molecular Plant Sciences, Institute of Plant Physiology and Ecology, Shanghai 200032, China; 5 University of Chinese Academy of Sciences, Beijing 100049, China

**Keywords:** silkworm, diapause, diapause hormone, RNA sequencing

## Abstract

**Simple Summary:**

Most insects species enter a diapause period to ensure their survival under unsuitable environments. In the silkworm, *Bombyx mori*, diapause occurs at the embryonic stage and is regulated by the diapause hormone (DH). Here, we directly injected DH into the female pupae and investigated the DH receptor expression pattern. Using RNA sequencing, we analyzed the gene expression profile in the developing ovaries after the injection of DH.

**Abstract:**

Diapause is a form of dormancy that organisms use to adapt to extreme environments by exhibiting developmental arrest. In the silkworm, *Bombyx mori*, diapause is thought to be elicited by diapause hormone (DH) signaling, which consists of interactions between DH and the DH receptor (DHR). However, the steps downstream of the DH signaling pathway are largely unknown. In the present study, we directly injected synthesized DH into the female pupae of a multivoltine, non-diapausing strain at 36 h after pupation. We found that the mRNA level of *DHR* declined at 4 h and recovered at 12 h after the injection of DH. Thus, we sequenced the transcriptome of the ovaries at 4 h and 12 h after the injection of DH. We identified 60 and 221 differentially expressed genes at 4 h and 12 h after the injection, respectively. All DEGs were identified, relating to 20E-related genes, JH-related genes, cellular detoxification, ribosomal proteins, lipid metabolism, and epigenetic modifications. Eleven genes were selected from the above categories to verify the transcriptome data. The qRT-PCR and RNA-Seq expression patterns of the genes were consistent, which indicated the authenticity and reliability of the transcriptome data. This study dramatically expands upon our knowledge of gene expression variation at the early phase of DH release.

## 1. Introduction

To ensure survival under adverse environmental conditions, most insect species have evolved a special stage of developmental arrest called diapause [1]. During the diapause phase, the insects show reduced respiration, an arrest of development, hormonal and metabolic mechanisms of change, and resistance to enhancement [2]. Diapause can occur during different developmental stages, including embryonic [3], larval [4], pupal [5], or adult stages [6].

The silkworm, *Bombyx mori*, is a typical insect that enter the diapause phase during the embryonic stage [7]. In natural conditions, *B. mori* strains can be divided into monovoltine, bivoltine, and multivoltine. Most silkworm strains experience one generation of breeding cycle (monovoltine) or two generations (bivoltine) per year [8]. Several tropical strains have evolved to be multivoltine and no longer experience diapause. When eggs are incubated under continuous darkness at 25 °C (25DD) or 15 °C (15DD), the resultant moths produce diapause or non-diapause eggs, respectively [8,9]. If the eggs are incubated at 20 °C under continuous illumination (20LL) or darkness (20DD), the resultant moths lay diapause or non-diapause eggs, respectively [10]. Thus, the diapause of the bivoltine strain is transgenerationally induced as a maternal effect, and is mainly determined by the environmental temperature and the photoperiod conditions during the embryonic development of the mother [11,12].

In *B. mori*, diapause is regulated by the diapause hormone (DH), a member of the FXPRLa neuropeptide family that is secreted and synthesized by the subesophageal ganglion (SG) [13]. DH released into the hemolymph and acts on ovary-expressed DH receptor (DHR) specificity, which induces the initiation of diapause [14]. Under the control of DH, a diapause-destined silkworm lays diapause eggs with certain changes, such as the color gradually changing from light yellow to brown within 48 h after oviposition [15] and cell cycle arrest the G2 stage [3]. Although DH is also expressed in insects other then *B. mori*, it is interesting that DH only effects diapause induction in *B. mori* [2]. In *Helicoverpa armigera*, the function of DH even terminates diapause [16,17].

In the bivoltine strain, the null mutants of either *DH* or *DHR* produce non-diapause eggs [12]. However, the overexpression of *DH* and *DHR* in the multivoltine silkworm is not enough to induce diapause, and they only cause changes in the gene expression that are related to diapause [18] The *Bombyx* TRPA1 ortholog (*BmTrpA1*) acts as a thermosensitive transient receptor potential channel that is activated at temperatures above ~21 °C, and that regulates progeny diapause via DH release [19]. Recently, the γ-aminobutyric acid (GABA)ergic and corazonin signaling system have been confirmed to modulate progeny diapause via diapause hormone release, which may be finely tuned by the temperature-dependent expression of GABA transporter via *BmTrpA1* [20]. 

The steps upstream of the DH signaling pathway are well-studied in silkworms. In contrast, the steps downstream of the DH signaling pathway are still unclear. In the diapause silkworm strains, the ovaries are sensitive to DH release during the period from 24 to 48 h of pupal age. Direct injection of DH at the sensitive period can induces diapause in a multivoltine strain, which is suitable for an analysis of the gene expression response of the ovaries to the stimulation of DH. In the present study, we injected the DH into the pupas of a multivoltine strain, Nistari, at 36 h, and performed RNASeq analysis.

## 2. Materials and Methods

### 2.1. Animals and Sample Preparation

A multivoltine and non-diapause silkworm strain, Nistari, was provided by JiangSu University of Science and Technology. Larvae were feed on fresh mulberry leaves under standard conditions at 25 °C with a 12 h light/dark cycle and 65–75% relative humidity. DH is a neuropeptide composed of 24 amino acids, and the sequence is as follows: Thr-Asp-Met-Lys-Asp-Glu-Ser-Asp-Arg Gly-A1a-His-Ser-Glu-Arg Gly-Ala-Leu-Cys-Phe-GIy-Pro-Arg-Leu-NH_2_ [7]. DH (sequence: TDMKDESDRGAHSERGALWFGPRL-NH_2_) was synthesized by Sangon Biotech (Shanghai, China). The injection was performed on female pupae at the intersegment membrane of the third abdominal segment at 36 h after pupation. The normal physiological titer of DH in *Bombyx mori* is 8.85 ± 2.71 pmol [12]. Cui et al. found that the diapause rate was approximately 100% only when the pupa was supplemented with 10 μg (3780 pmol) of DH [21]. In the experimental group, the female pupae were injected with 2 μL DH (5 μg/μL) in order to achieve the best diapause effect. In control group, the female pupae were injected with 2 μL UltraPure™ DNase/RNase-Free distilled water (Invitrogen, Waltham, CA, USA). The silkworm pupae were placed in the same growing environment as the larval stage, and then mated and laid eggs as adults. The silkworm eggs were incubated in darkness at 25 °C, and the diapause rate of 15 egg circles was calculated 48 h after oviposition. Female pupae were anesthetized on ice for 30 min, and then ovarian tissue was collected in phosphate-buffered saline after injection at 4 h and 12 h. The ovaries of three silkworms were collected from each sample. The samples were quickly frozen in liquid nitrogen, and stored at −80 °C for RNA extraction.

### 2.2. RNA Extraction and cDNA Library Construction 

Total RNA was extracted with TRIzol reagent (Invitrogen, Waltham, CA, USA) and digested with DNase I (Invitrogen, Waltham, CA, USA) according to the manufacturer’s protocol. RNA quality was determined using a NanoDrop ND-1000 Spectrophotometer and agarose gel electrophoresis (NanoDrop, Wilmington, DE, USA) (Figure S1). The cDNA libraries were constructed from the quantified total RNA using a TruSeq™ RNA Sample Preparation kit (Illumina, San Diego, CA, USA), following the manufacturer’s recommendations. Three biological replications were performed with each treatment.

### 2.3. RNA Sequencing (RNA-Seq) and De Novo Assembly of Sequences

The constructed cDNA library was sequencing on an Illumina HiSeq™ 2000 platform (Mega, Shanghai, China). Low-quality reads, reads with adapters, and unknown nucleotides were removed from the raw reads prior to assembly, to obtain clean reads. Low-quality reads, reads with adapters, and unknown nucleotides were removed from the raw reads prior to assembly, to obtain clean reads. The reference genome was downloaded from the SilkBase website (https://silkdb.bioinfotoolkits.net/main/species-info/-1, accessed on 16 July 2019). The high-quality clean reads were de novo reassembled by Trinity software [22].

### 2.4. Differentially Expressed Genes (DEGs) Analysis

Gene expression levels were calculated by the FPKM [23] (the expected number of fragments per kilobase of transcript sequence per millions of base pairs sequenced) method, based on the length of the gene and the gene length for the read counts. Differential expression analysis between the treatment and control groups (three biological replicates per condition and the ovaries of three silkworms were collected per sample) was performed using DESeq software [24]. Genes with an adjusted *p*-adjust of <0.05 and a |log2 (fold change)| > 1 as found by DESeq2 were assigned as differentially expressed. To determine the main biological functions of DEGs, Blast2GO software was used to annotate them with GO terms [25]. The GO enrichment analysis of DEGs was implemented by Goatools software [26]. Corrected GO terms with *p*-adjust < 0.05 were considered to be significantly enriched in DEGs. To explore the biological interactions between the DEGs, KEGG pathways were enriched using KOBAS software and corrected using hypergeometric tests and the Benjamini–Hochberg false discovery rate (FDR) [27]. 

### 2.5. Quantitative Real-Time PCR (qRT-PCR) Verification 

To verify the accuracy of the RNA-seq, a total of 11 genes were selected for qRT-PCR verification. Total RNA was isolated from the ovaries using a TRIzol reagent (Invitrogen). The RNA was treated with DNase I (Invitrogen) to remove genomic DNA. One μg of total RNA was used to synthesize cDNA using the ReverAid First Strand cDNA Synthesis Kit (Fermentas). Relative mRNA levels were determined by qRT-PCR using SYBR Green real-time PCR master mix (TOYOBO, Osaka, Japan). The PCR conditions used were as follows: initial incubation at 95 °C for 1 min, followed by 40 cycles of 95 °C for 15 s, and 60 °C for 1 min. The 2^−^^∆∆^^CT^ method was employed to calculate relative gene expression, and the data were normalized to *BmRP49*. The primers used for qRT-PCR are listed in Table S1.

### 2.6. Statistical Analysis 

The normality of data distribution was checked using the Shapiro–Wilk test. Experimental data from multiple groups were analyzed using a one-way analysis of variance with the Bonferroni post hoc test. Experimental data from two groups were analyzed using a Student’s *t*-test (*, *p* < 0.05; **, *p* < 0.01; ***, *p* < 0.001). At least three independent replicates were used for each treatment, and the data are reported as means ± standard errors of the mean (SEM).

## 3. Results

### 3.1. The Injection of DH Induces Progeny Embryonic Diapause in a Non-Diapause Silkworm Strain 

**Figure 1:**
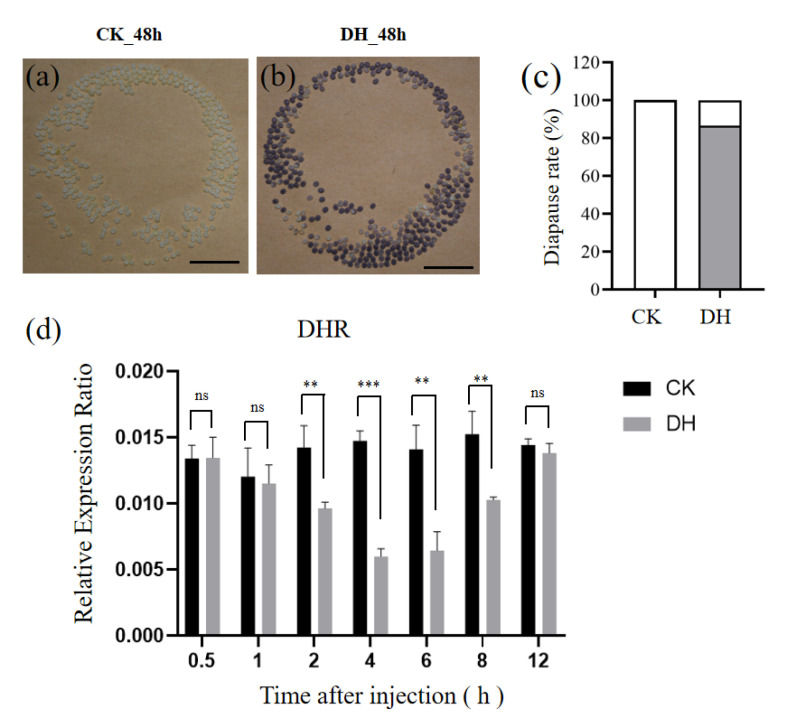
DH induces diapause in the Nistari strain. (**a**–**c**) The typical egg batches 48 h after oviposition from each distilled water-injected (CK_48h) and DH-injected (DH_48h) silkworm in the Nistari strain. Scale bar: 1 cm. (**d**) The expression patterns of *BmDHR*. qRT-PCR was used to detect the expression levels of *BmDHR* gene in the ovary at 0.5 h, 1 h, 2 h, 4 h, 6 h, 8 h, and 12 h. *B. mori* ribosomal protein gene *RP49* was used as a control for normalization. The data shown are mean values ± S.E.M. (*n* = 3). The asterisks indicate the significant differences with a two-tailed *t*-test, ** *p* < 0.01; *** *p* < 0.0001. The ns indicate the no significant differences.

To characterize the effect of DH, which induces the occurrence of silkworm egg diapause, on a non-diapause silkworm strain, Nistari, the female pupae were injected with synthetic DH (10 µg) at a pupal age of 36 h. In the control group, the eggs laid by the moths injected with distilled water (CK) were all light yellow non-diapause eggs (Figure 1a). In contrast, we found that the percentage of brown diapause eggs laid by the moths injected with DH reached up to 86.6% (Figure 1b,c). These results indicate that a direct injection of DH can induce diapause in the Nistari strain. In the diapause strains, DH acts on the DHR specificity expressed in ovary, which induces the initiation of diapause [13]. Thus, we detected the mRNA expression level of *DHR* after the DH injection. We found that the mRNA level of *DHR* gradually declined from 2 h to 4 h, and it recovered at 12 h after the injection of DH (Figure 1d and Figure S2). The change trend of *DHR* in female pupae after DH injection from 0.5 h to 12 h suggests that 4 h and 12 h might be the key point of DH action. In order to understand the physiological and biochemical effects of DH binding to DHR on oocytes, ovarian tissues at 4 h and 12 h after DH injection were collected for transcriptome sequencing.

### 3.2. Overview of Transcriptome Sequencing Data

Based on the expression level of *DHR*, we sequenced the transcriptomes of the ovaries at 4 h and 12 h after the injection of DH. The 12 independent cDNA libraries constructed from the control group treated with distilled water (CK) and from the experiment group treated with DH were sequenced on the Illumina HiSeq™ 2000 platform. The clean data of each sample was more than 6.06 Gb, and the percentage of Q20 and Q30 bases was more than 97% and 93%, respectively. The GC values of all samples ranged from 45% to 46% (Table 1). The result showed that the sequencing results were accurate and reliable, and could be used for subsequent analysis. PCA analysis showed a clear spatial separation of the gene expression profiles for the control and DH treatments (Figure S3).

### 3.3. DH-Induced Transcriptional Changes

**Figure 2:**
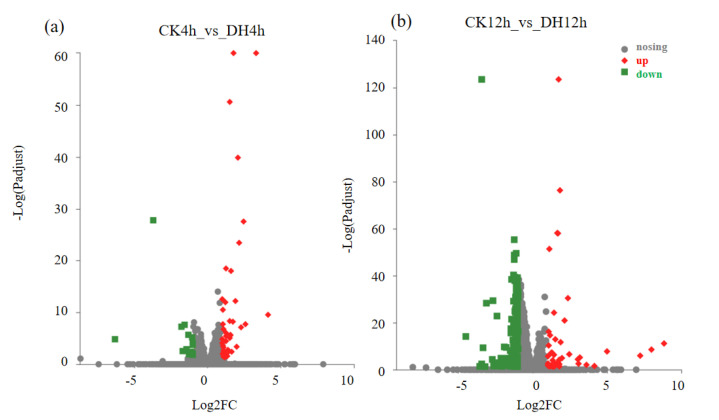
Volcano plot of DEGs identified in ovaries at 4 h (**a**) and 12 h (**b**) after DH injection. *X*-axis: log2−fold change (treatment/control). *Y*-axis: log10(FDR). Red data points indicated up-regulated unigenes and green data points indicated down-regulated unigenes.

When considering the *p*-adjust < 0.05 and a |log2 (fold change)| > 1, 259 differentially expressed genes (DEGs) were found at 4 h and 12 h after DH injection. Among the 259 DEGs, there were 60 and 221 genes that were differentially expressed at 4 h and 12 h after DH injection, respectively (Figure 2a,b; Tables S2 and S3). It is worth noting that the number of DEGs showed an upward trend, which indicated that the effect of DH on the ovaries increased gradually. Furthermore, 22 were differentially expressed in both periods (common), while 38 (4 h-specific) and 199 (12 h-specific) were only differentially expressed at 4 h and 12 h after DH injection, respectively (Figure 2b). DH led to significant changes in five times as many genes at 12 h than at 4 h. The DH treatment after 12 h significantly elicited more down-regulated genes, with more than half of the DEGs in both phases being down-regulated compared with the control larvae. 

**Figure 3:**
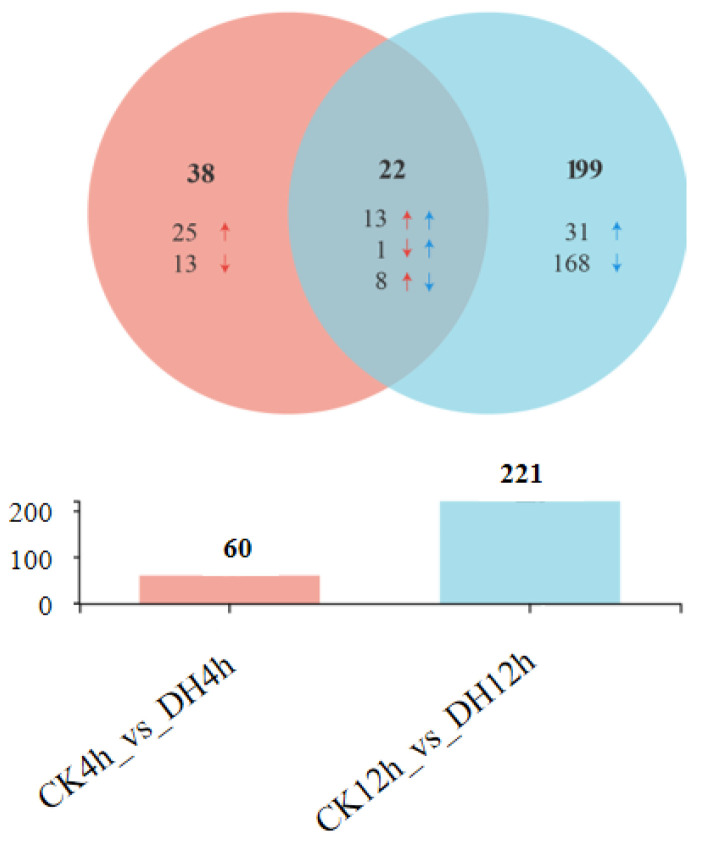
Number of differentially regulated genes in response to DH. Numbers of DEGs (|FC| > 2 and *p*-adjust < 0.05) between control and DH-treated for 4 h and 12 h. The circles of different colors represent the genes in a gene set, where the value represents the number of genes shared or unique between CK4h_vs._DH4h and CK12h_vs._DH12h.

We further analyzed the 22 differentially expressed in both period after DH injection (Figure 3). The common genes can be divided into three categories. In the first category, compared with the control group, 13 genes were up-regulated in the DH-treated group at both 4 h and 12 h after injection, including *Juvenile hormone binding protein* (*JHBP*), *fatty acyl-CoA reductase (Wat), cytochrome P450 CYP4G25 (CYP4G25), heparan sulfate 2-O-sulfotransferase pipe (pip)*, *UDP-glycosyltransferases (UGT2A3) and Glutathione S-transferase* (*GST1*), *serine protease inhibitor 11 precursor (Spn42Dd),* and *diacylglycerol kinase eta-like*, etc. In the second category, compared with the control group, the DH-treated group only had one gene: *WD repeat-containing protein 18* (*Wdr18*), which was down-regulated at 4 h after injection but up-regulated at 12 h after injection. In the third category, compared with the control group, eight genes in the DH-treated group were up-regulated at 4 h after injection but down-regulated at 12 h after injection, including *Broad* (*br*), *single-minded homolog 2*(*Ahr*), *reticulon-1-A isoform X2*(*rtn1-a*), *histone H3(His3)*, *serine proteinase-like protein*(*PPAF2*), and *endochitinase A isoform X1,* etc. 

### 3.4. Gene Ontology (GO) Classification of DEGs

**Figure 4:**
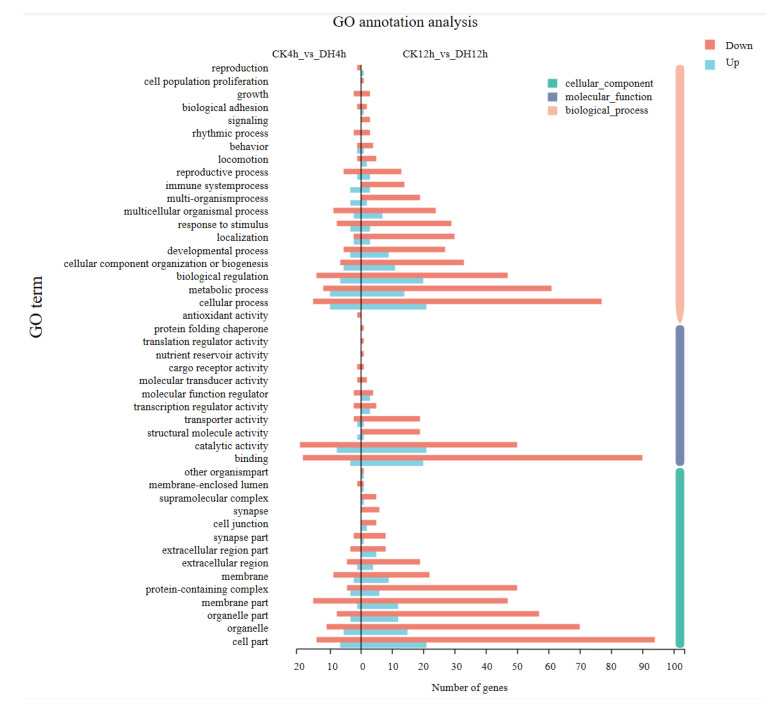
GO annotation analysis of DEGs in CK4h_vs._DH4h group and CK12h_vs._DH12h group. The vertical axis represents number of DGEs mapped to indicated GO term, and the horizontal axis represents each GO term. Red and blue bars represent up-regulated and down-regulated genes, respectively.

**Figure 5:**
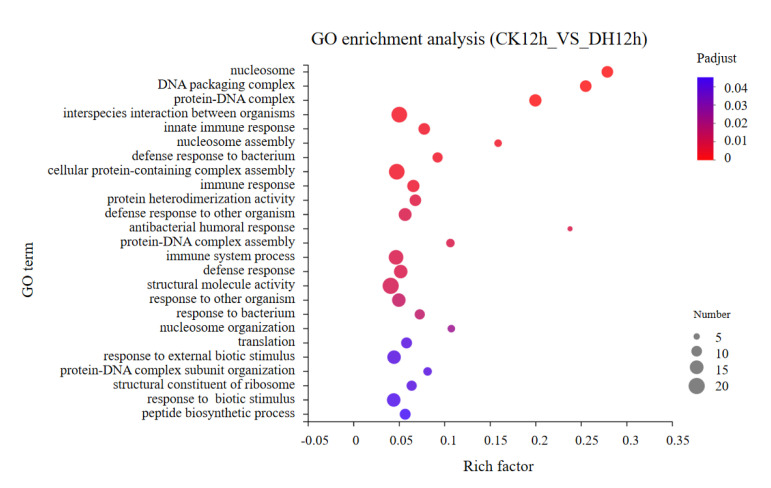
GO enrichment of DEGs in CK12h_vs._DH12h group. The vertical axis represents GO term and the horizontal axis represents rich factor. A greater rich factor indicates a greater degree enrichment. The size of the plot represents the number of DEGs in this GO term. The color of the plot corresponds to different *p*−adjust ranges.

To investigate the functional significance of the DEGs identified above, GO function annotations and enrichment analysis were executed. All DGEs were classified into three main GO categories: biological process, cellular component, and molecular function, and then to 45 functional categories. In these functional groups, GO terms in CK4h_vs._DH4h were similar to CK12h_vs._DH12h (Figure 4 and Table S4). GO term enrichment analysis showed that when considering *p*-adjust < 0.05, none of the GO terms were significantly enriched between CK_4h and DH_4h. The DEGs between CK_12h and DH_12h were significantly enriched for 25 GO terms (Figure 5 and Table S5). The majority of the DEGs were engaged in the biological process, including immune response. In the cellular component group, the DEGs were all involved in chromatin formation, including nucleosome, DNA packaging complex, and protein–DNA complex. In the molecular function group, the majority of DEGs were engaged in protein heterodimerization activity, structural molecule activity, and structural constituent of ribosome.

### 3.5. KEGG Enrichments Analysis of DEGs

**Figure 6:**
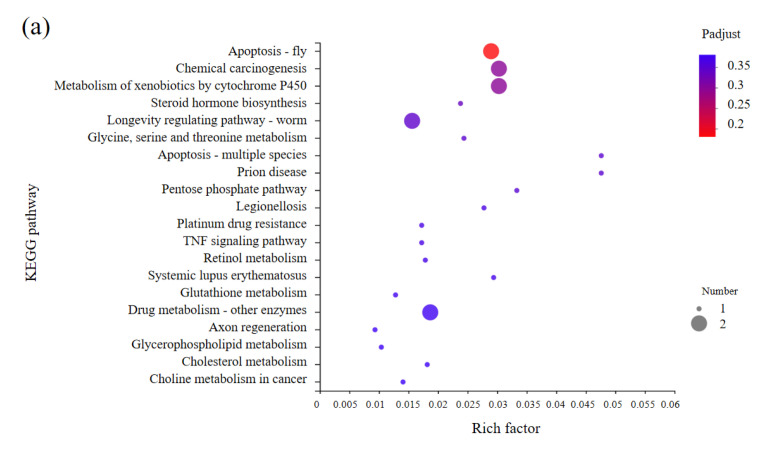

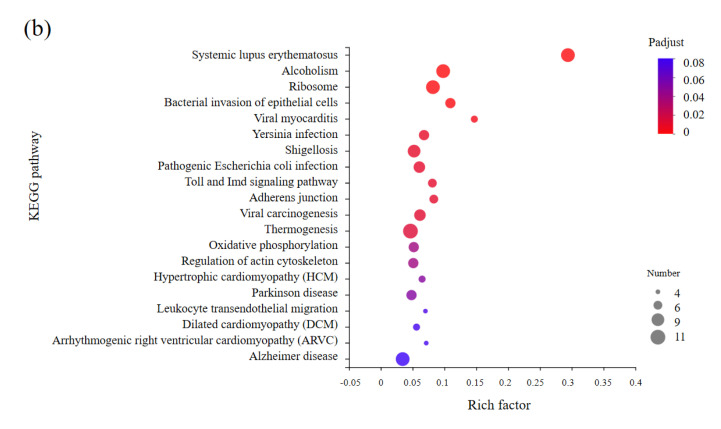
Top 20 pathways of KEGG enrichments analysis. (**a**) DEGs in CK4h_vs._DH4h group; (**b**) DEGs in CK12h_vs._DH12h group. The vertical axis represents pathway name and the horizontal axis represents rich factor. A greater rich factor indicates a greater degree enrichment. The size of the plot represents the number of DEGs in this pathway. The color of the plot corresponds to different *p*−adjust ranges.

To investigate the influence of metabolism pathways after DH injection between 4 h and 12 h, we carried out KEGG enrichment analysis for DEGs in CK4h_vs._DH4h group (Table S6) and CK12h_vs._DH12h group (Table S7). The top 20 abundant pathways were shown in Figure 6. The result showed that DEGs in CK4h_vs._DH4h group were mostly enriched in apoptosis, which were related to cell growth and death. In addition, several pathways related to metabolism were also enriched, like metabolism of xenobiotics by cytochrome P450, steroid hormone biosynthesis and glycine, serine and threonine metabolism et al. (Figure 6a). However, DEGs in CK12h_vs._DH12h were also enriched in a variety of immune related pathways, such as toll and imd signaling pathway and leukocyte transendothelial migration (Figure 6b). These results indicated that inject DH can affect the metabolism and immune of early ovary development.

### 3.6. Validation of DEGs by qRT-PCR

**Figure 7:**
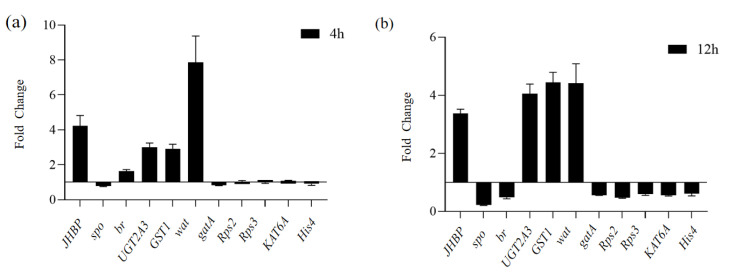
Quantitative real-time PCR analysis of candidate gene expression of ovaries induced by DH after 4 h (**a**) and 12 h (**b**). The vertical axis represents the -fold change of candidate genes in DH group compare with CK group.

In order to confirm the reliability of the RNA-Seq data in the present study, the 11 genes including *JHBP*, *spo (spook)*, *br, UGT2A3, GST1*, *Wat*, *gatA (fatty-acid amide hydrolase 2-like), Rps2 (ribosomal protein S2*), *Rps3 (ribosomal protein S3*), *KAT6 (histone acetyltransferase KAT6A isoform X1),* and *His4 (histone H4)* were selected to perform qRT-PCR. The results showed that the qRT-PCR and RNA-Seq expression patterns of these 11 differential genes were consistent (Figure 7 and Figure S4), indicating that the transcriptome data are authentic and reliable, and can be used for subsequent analysis. The 11 DEGs showed remarkable differences, which are also consistent with the results obtained from the RNA sequencing data.

## 4. Discussion

Insects enter diapause to ensure their survival under unfavorable environmental conditions, often under the control of photoperiod, humidity, and temperature [2,28]. In *B. mori*, the photoperiod and temperature of embryonic development regulate the release of DH at the middle pupal stages, during which the developing ovaries are sensitive to DH [29]. The released DH acts on DHR that is expressed in the developing ovaries, which induces the initiation of embryonic diapause. In the present study, gene expression changes were compared in the developing ovaries after the induction of DH by RNAseq. We identified 60 DEGs 4 h after DH injection compared with CK, of which 46 were up-regulated and 14 down-regulated. At 12 h after DH injection, 221 DEGs were identified compared with CK, with 45 up-regulated and 176 down-regulated. GO classification and KEGG enrichment analysis showed that most of the DEGs were involved in ribosomal proteins, epigenetic modification, metabolism process, and immune response. At the same time, we focused on hormone-related genes in DEGs, such as 20E-related genes and JH-related genes.

Diapause is induced by various hormones and peptides in different insect species [30,31]. In *Helicoverpa armigera*, pupal diapause is mainly regulated by the molting hormone 20-hydroxyecdysone (20E) [32]. A previous study showed that the low titer of ecdysteroids may be involved in the induction of embryonic diapause in *B. mori* [33]. Currently, we find that a Halloween gene, *spo*, is involved in 20E biosynthesis, which was significantly down-regulated 12 h after DH injection in the ovaries. Earlier studies on *Drosophila melanogaster* and *B. mori* showed that the *Spo* worked in a black box [34,35]. *Spo* is expressed in the prothoracic gland (PG) of *B. mori*, and its transcriptional peak is consistent with the increase of 20E secretion [34]. In female mosquitoes, transient *spo* knockout resulted in the decreased secretion of 20E in the ovaries, suggesting that its secretion was related to *spo* [36]. Therefore, we speculated that the down-regulated expression of *spo* in the ovarian tissues of the DH injection group would lead to the involvement of 20E in the induction of diapause in the offspring of the silkworm. Therefore, we hypothesized that *spo* also participates in diapause induction at an early pupal stage by controlling the synthesis of 20E. Br, a vital regulator factor of the ecdysone cascade, plays crucial roles during insect growth processes [37]. Br is also involved in the regulation of reproduction in many insects, such as the cockroach *Blattella germanica* and the mosquito *Aedes aegypti* [38,39]. It has been reported that br can directly regulate the transcription of *vitellogenin* (vg) to control vitellogenesis and egg formation through the transmission of ecdysone signal in *B. mori* [40]. In this study, we found that the expression level of *Br* was significantly up-regulated at 4 h, and down-regulated at 12 h after DH injection. The results suggest that the 20E cascade was involved in inducing progeny diapause by DH injection in the silkworm pupal stage.

Insect growth and development are orchestrated by 20E and juvenile hormone (JH) [41,42]. In addition to the gene expression changes involved in the 20E cascade, the JH-related genes expression level was also affected after the induction of DH. Our results showed that JHBP was significantly up-regulated in the DH group, compared with the control group. The function of JHBP is to bind JH in hemolymph to protect JH from nonspecific degradation, and to transport hormones to target tissues [43,44]. There are few reports regarding JH in the insect pupal stage, and most studies focus on the larvae and adults. In the larval stage, JH can maintain a larval state after molting. In the adult stage, JH can directly regulate reproductive maturation [45], such as *Locusta migratoria* [46]. It is generally believed that JH is low in pupal insects. In *Philosamia cymhia ricini*, the JH titers in the pupal stage were gradually increased [47]. JH titers in the hemolymph of *Galleria mellonella* was generally low in the pupal stage, but they increased drastically in the late pupal stage [48]. Similarly, JH was also expressed in the pupal stage of *Bombyx mori* [49]. Studies have shown that injecting juvenile hormone analogue (JHA) into female pupae can affect the maturation of silkworm eggs [50]. Our results suggest that JHBP may also be involved in inducing progeny diapause by DH injection in the silkworm pupal stage.

Ribosomal proteins (Rp) are mainly involved in protein synthesis, cell metabolism, and other important physiological functions, including ovarian and embryonic development [51,52,53,54]. In this study, we found that 10 DEGs involved in ribosome biogenesis were significantly down-regulated in CK_12h. In *Culex pipiens*, the RNA interference of *Rps3a* can mimic “diapause-like” ovarian arrest in non-diapause females [55]. In *Drosophila*, a *string of pearls (SOP)* encoding Rps2 is necessary for successful oogenesis [56]. The silencing of *RpS2* induces diapause in adult females of *C. pipiens* [30]. In addition, the ribosomal protein in diapause eggs was lower than that in non-diapause eggs in *B. mori* [57]. Therefore, we speculated that the downregulation of genes involved in ribosome biogenesis was responsible for the low content of ribosomal protein in diapause eggs. However, detailed functions remain to be investigated.

Lipid metabolism is critical for energy homeostasis [58]. We noticed that *fatty acyl-CoA reductase* (Wat) was significantly up-regulated in DH_4h and DH_12h. Wat plays an important role in the formation of long-chain fatty alcohols, which are precursors of insect wax lipids and ether lipids. Wax lipids can reduce water evaporation and enhance defenses against microorganisms; ether lipids are important membrane components [59,60]. We speculated that the up-regulation of *Wat* expression contributes to the enhanced stress resistance of silkworms.

UDP-glycosyltransferase (UGT) and Glutathione S-transferase (GST) play important roles in cellular detoxification in insects [61,62]. In DH_4h and DH_12h, the expressions of *UGT2A3* and *GST1* were significantly up-regulated. UGT protects cellular systems from toxic compounds by catalyzing the binding of a sugar provided by UDP-glycosides to lipophilic molecules to produce water-soluble products that are easily excreted [63]. On the other hand, GST catalyzes the reaction between reduced glutathione (GSH) and toxic substances to reduce their toxicity and to enhance their dissolubility and excretion, thus achieving the purpose of detoxification [64]. In some diapause insects, such as *Trichogramma dendrolimi*, the expression levels of *UGT* and *GST* are also increased to enhance resistance to adverse environmental conditions [65]. Thus, the high expression of *UGT2A3* and *GST1* in the developing ovaries may also be associated with improved stress resistance. 

The immune system remains active at diapause [66]. In diapausing Colorado potato beetle, several serine protease transcripts in the immune response were up-regulated in both the fat body and the flight muscle [67]. Genes involved in the immune response were up-regulated during diapause in *Tetrapedia diversipes* [68]. In our study, we found that various DEGs involved in the immune response were affected in DH_12h. This result suggests that the immune response may play an important role in the diapause of *B. mori* induced by DH. 

Epigenetic modification refers to changes in gene expression levels through DNA methylation, small regulator RNA, and histone acetylation, without changing the nucleotide sequences of genes [69]. Epigenetic modification has recently been proven to play an important role in diapause induction in many insects [70,71,72]. In *Sarcophaga bullata*, the total *histone H3 acetylation* was significantly reduced during the diapause period. The expression levels of *histone acetyltransferases* and *histone deacetylases* were changed at pre-diapause, diapause, and post-diapause periods, supporting histone modifications involved in diapause regulation [72]. In this study, we found 11 histones and one histone acetyltransferase KAT6A in the set of down-regulated gene in CK12h_vs._DH12h, indicating that histone modifications are involved in the regulation process of DH-induced diapause in silkworms.

## 5. Conclusions

We injected synthetic DH into the female multivoltine silkworm at 36 h after pupation. According to the expression level of *DHR*, ovarian tissues at 4 h and 12 h after the injection of DH were collected for transcriptome sequencing. GO analysis and KEGG enrichment analysis were performed on the DEGs, which could be divided into the following categories according to their biological functions: 20E-related genes, JH-related genes, cellular detoxification, ribosomal proteins, lipid metabolism, and epigenetic modifications. Eleven genes were selected from the above categories to verify the transcriptome data. Our results provide important information for the steps downstream of the DH signaling pathway, and they have important guiding significance for the further study of diapause in *Bombyx mori*.

## Figures and Tables

**Table 1:** Summary of the transcriptome sequencing data from the control and DH-treated samples.

Sample	Raw Reads	Clean Reads	Error Rate (%)	Q20 (%)	Q30 (%)	GC Content (%)
DH12h_3	42,895,926	42,586,708	0.0259	97.59	93.33	45.15
DH12h_2	42,493,302	42,166,558	0.0258	97.66	93.49	44.97
DH12h_1	43,438,602	43,116,974	0.0261	97.53	93.15	45.14
CK12h_3	42,469,504	42,131,634	0.0255	97.77	93.76	46.18
CK12h_2	46,026,052	45,682,102	0.0256	97.72	93.61	46.68
CK12h_1	45,596,578	45,186,554	0.026	97.57	93.3	46.73
DH4h_3	44,528,428	44,154,426	0.0256	97.72	93.62	45.96
DH4h_2	44,029,406	43,654,302	0.0258	97.63	93.43	45.94
DH4h_1	41,634,522	41,229,916	0.026	97.56	93.32	46.29
CK4h_3	44,979,162	44,663,354	0.0255	97.78	93.73	45.02
CK4h_2	46,506,402	46,104,824	0.0263	97.43	92.96	45.5
CK4h_1	44,441,656	44,079,890	0.026	97.56	93.27	45.46

## Data Availability

The data presented in this study are available on request from the corresponding author.

## Supplementary Materials

The following supporting information can be downloaded at: https://www.mdpi.com/article/10.3390/biology11091313/s1, Figure S1: Agarose gel electrophoresis diagram of RNA quality detection; Figure S2: The expression pattern of *BmDHR*; Figure S3: PCA analysis; Figure S4: RNA-Seq expression patterns; Table S1: Primers of the genes for qRT-PCR validation in this study; Table S2: Differentially expressed genes (DEGs) among CK4h_vs._DH4h; Table S3: Differentially expressed genes (DEGs) among CK12h_vs._DH12h; Table S4: GO annotation analysis of DEGs in CK4h_vs._DH4h group and CK12h_vs._DH12h group; Table S5: GO enrichment of DEGs in CK12h_vs._DH12h group; Table S6: KEGG enrichment of DEGs from different groups in CK4h_vs._DH4h; Table S7: KEGG enrichment of DEGs from different groups in CK12h_vs._DH12h.
