# Supplementary material for: Transcriptional Dynamics Induced by Diapause Hormone in the Silkworm, Bombyx mori
Source: Biology (Basel). 2022 Sep 4;11(9):1313. doi: 10.3390/biology11091313 (PMC9495520; doi:10.3390/biology11091313)
Supplement: Supplementary file 1 [file biology-11-01313-s001.zip › Supplementary Materials figure.pdf]

Supplementary Materials: Diapause hormone induced diapause revealed by RNAseq in multivoltine silkworm, *Bombyx mori*

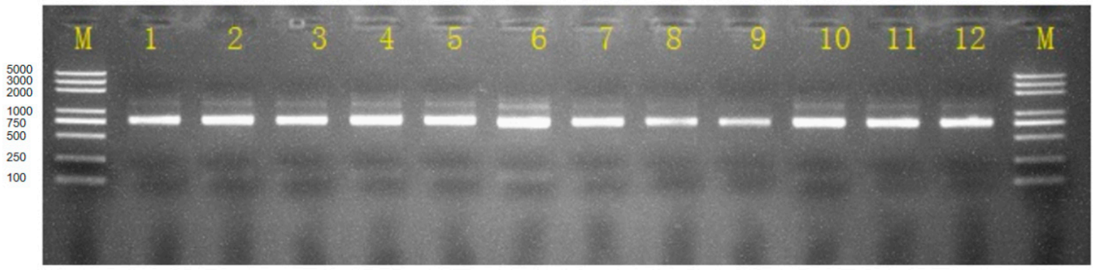

Figure S1. Agarose gel electrophoresis diagram of RNA quality detection. 1-12: CK_4h-1, CK_4h-2, CK_4h-3, DH_4h-1, DH_4h-2, DH_4h-3, CK_12h-1, CK_12h-2, CK_12h-3, DH_12h-1, DH_12h-2, DH_12h-3. M: marker.

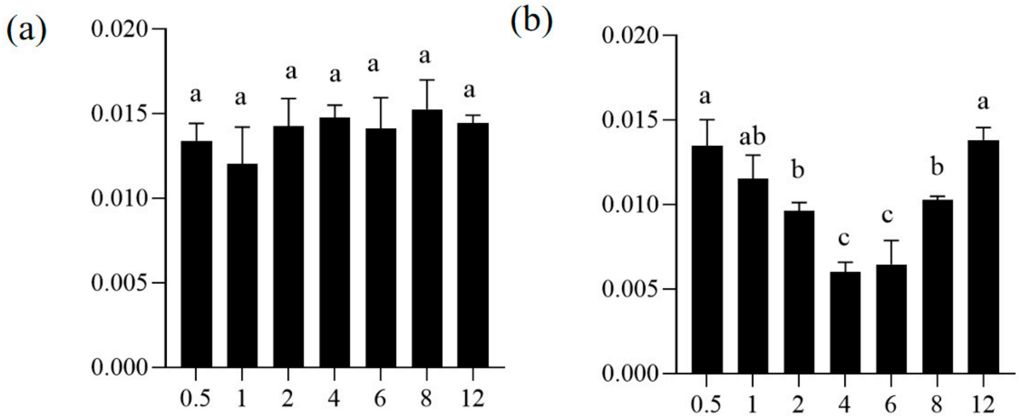

Figure S2. The expression pattern of *BmDHR*. qRT-PCR was used to detect the expression level of *BmDHR* gene in the ovary at 0.5h, 1h, 2h, 4h, 6h, 8h and 12h. *B. mori* ribosomal protein gene *RP49* was used as a control for normalisation. The data shown are mean values ± S.E.M. (n = 3). The data were analyzed by one-way analysis. Letters a, b, and c denote significant difference at $P<0.05$.

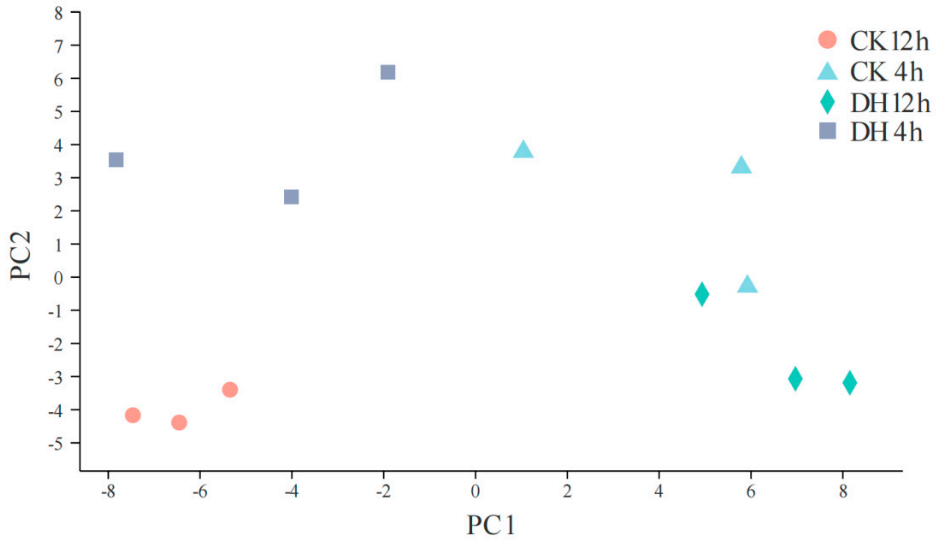

Figure S3. PCA analysis. X-axis: the contribution degree of principal component 1 (PC1) in the two-dimensional graph to the differentiated samples, and Y-axis: the contribution degree of principal component 2 (PC2) in the two-dimensional graph to the differentiated samples. The distance of each sample point represents the distance of the sample, and the closer the distance is, the higher the similarity between samples is.

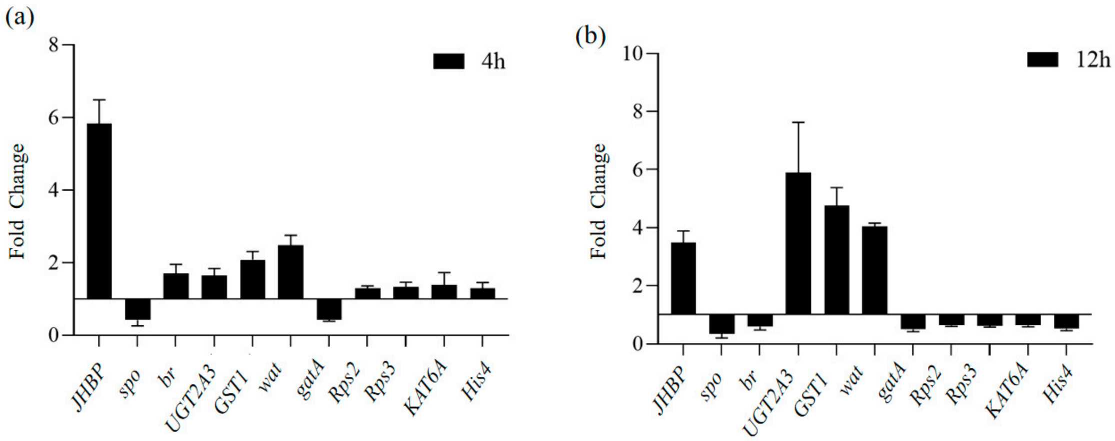

Figure S4. RNAseq of candidate gene expression of ovaries induced by DH after 4h **(a)** and 12h **(b)**. The vertical axis represents the fold change of candidate gene in DH group compare with CK group.
